# 2-Ethyl-6-methyl­anilinium 4-methyl­benzene­sulfonate

**DOI:** 10.1107/S160053680900230X

**Published:** 2009-01-23

**Authors:** Tian-Quan Wu, Lin Xia, Ai-Xi Hu, Jiao Ye

**Affiliations:** a College of Chemistry and Chemical Engineering, Hunan University, Changsha 410082, People’s Republic of China

## Abstract

The title compound, C_9_H_14_N^+^·C_7_H_7_SO_3_^−^, contains a 2-ethyl-6-methyl­anilinium cation and a 4-methyl­benzene­sulfonic anion. The cations are anchored between the anions through N—H⋯O hydrogen bonds. Electrostatic and van der Waals inter­actions, as well as hydrogen bonds, maintain the structural cohesion.

## Related literature

For related structures, see: Benali-Cherif *et al.* (2007 ▶); Benslimane *et al.* (2007 ▶); Elmali *et al.* (2001 ▶); Fábry *et al.* (2001 ▶, 2002 ▶); Khemiri *et al.* (2008 ▶); Muthamizhchelvan *et al.* (2005 ▶); Smirani *et al.* (2008 ▶); Smirani & Rzaigui (2009 ▶).
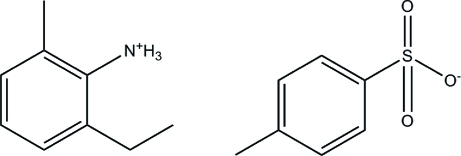


## Experimental

### 

#### Crystal data

C_9_H_14_N^+^·C_7_H_7_O_3_S^−^
*M* *_r_* = 307.40
Monoclinic, 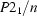

*a* = 15.2514 (9) Å
*b* = 6.1889 (4) Å
*c* = 16.9242 (10) Å
β = 102.850 (1)°
*V* = 1557.46 (16) Å^3^
*Z* = 4
Mo *K*α radiation
μ = 0.22 mm^−1^
*T* = 173 (2) K
0.45 × 0.34 × 0.27 mm

#### Data collection

Bruker SMART 1000 CCD diffractometer
Absorption correction: multi-scan (*SADABS*; Sheldrick, 2004 ▶) *T*_min_ = 0.904, *T*_max_ = 0.944
7882 measured reflections
3354 independent reflections
2555 reflections with *I* > 2σ(*I*)
*R*_int_ = 0.023

#### Refinement

*R*[*F* ^2^ > 2σ(*F* ^2^)] = 0.038
*wR*(*F* ^2^) = 0.109
*S* = 1.08
3354 reflections
194 parameters
H-atom parameters constrained
Δρ_max_ = 0.33 e Å^−3^
Δρ_min_ = −0.34 e Å^−3^

### 

Data collection: *SMART* (Bruker, 2001 ▶); cell refinement: *SAINT-Plus* (Bruker, 2003 ▶); data reduction: *SAINT-Plus*; program(s) used to solve structure: *SHELXS97* (Sheldrick, 2008 ▶); program(s) used to refine structure: *SHELXL97* (Sheldrick, 2008 ▶); molecular graphics: *SHELXTL* (Sheldrick, 2008 ▶); software used to prepare material for publication: *SHELXTL*.

## Supplementary Material

Crystal structure: contains datablocks I, global. DOI: 10.1107/S160053680900230X/pv2133sup1.cif

Structure factors: contains datablocks I. DOI: 10.1107/S160053680900230X/pv2133Isup2.hkl

Additional supplementary materials:  crystallographic information; 3D view; checkCIF report

## Figures and Tables

**Table 1:** Hydrogen-bond geometry (Å, °)

*D*—H⋯*A*	*D*—H	H⋯*A*	*D*⋯*A*	*D*—H⋯*A*
N1—H1*C*⋯O1^i^	0.91	1.88	2.7727 (18)	168
N1—H1*B*⋯O3^ii^	0.91	1.89	2.7800 (19)	166
N1—H1*A*⋯O2^iii^	0.91	1.95	2.7917 (18)	153

Symmetry codes: (i) 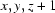; (ii) 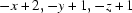; (iii) 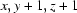.

**Fig. 1.**
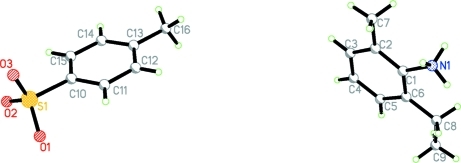
Molecular structure of the title compound showing 30% probability displacement ellipsoids.

**Fig. 2.**
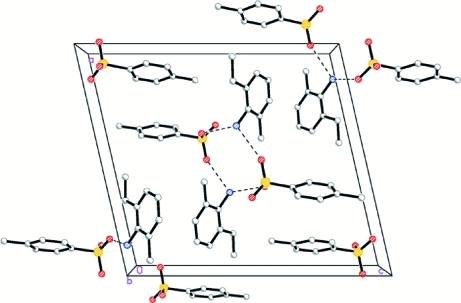
Unit cell packing diagram of (I) showing intermolecular hydrogen bonds; H atoms have been omitted for clarity.

## supplementary crystallographic information

### Comment

In the title compound, the proton of the sulfonic group of sulfonic acid has been transferred to the N atom of the 2-ethyl-6-methylaniline molecule, leading to the formation of the molecular complex, (I). In this article we present the crystal structure of the title compound. The removal of the sulfonic acid H atom leads to a shortening of the C10—S1 bond length, showing a partial double-bond character. This behaviour is similar to that observed in many picrate salts (Muthamizhchelvan *et al.* 2005); it is attributed to the loss of the hydroxyl proton, leading to the conversion of the neutral to an anionic state of the molecule.

In the structure (Fig. 1), intermolecular hydrogen bonds are observed, with the N atom of the cation acting as donors. The orientation of the anion and cation facilitates the formation of the expected strong N—H···O hydrogen bonds between amino atom N1 and the sulfonic O atoms; N1 hydrogen bonds to two sulfonic O atoms of adjacent molecules (Fig. 2 and Table 1). These hydrogen bonds are effective in the stabilization of the structure. The crystal structures of several related compounds have been published, e.g. Muthamizhchelvan *et al.* (2008); Benslimane *et al.* (2007); Smirani & Rzaigui (2009); Fábry *et al.* (2001, 2002); Khemiri *et al.* (2008); Benali-Cherif *et al.* (2007); Elmali *et al.* (2001).

### Experimental

Added 2-ethyl-6-methylaniline (1.35 g) into a solution of 4-methylbenzenesulfonic acid (1.72 g) and ethanol (10 ml). After 10 min precipitate were formed which were filtered and dried, giving the desired product. Crystals suitable for X-ray structure determination were obtained by slow evaporation of an ethanol solution at room temperature.

### Refinement

The methyl H atom were positioned geometrically (C—H = 0.98 Å) and torsion angles refined to fit the electron density [U_iso_(H) = 1.5U_eq_(C)]. Other H atoms were placed at calculated positions (methylene C—H = 0.95 Å and aromatic C—H = 0.95 Å) and included in the refinement in a riding mode with [U_iso_(H) = 1.2U_eq_(C)].

### Crystal data

**Table:**

C_9_H_14_N^+^·C_7_H_7_O_3_S^−^	*F*(000) = 656
*M**_r_* = 307.40	*D*_x_ = 1.311 Mg m^−^^3^
Monoclinic, *P*2_1_/*n*	Melting point: 428 K
Hall symbol: -P 2yn	Mo *K*α radiation, λ = 0.71073 Å
*a* = 15.2514 (9) Å	Cell parameters from 3656 reflections
*b* = 6.1889 (4) Å	θ = 2.5–27.0°
*c* = 16.9242 (10) Å	µ = 0.22 mm^−^^1^
β = 102.850 (1)°	*T* = 173 K
*V* = 1557.46 (16) Å^3^	Block, colourless
*Z* = 4	0.45 × 0.34 × 0.27 mm

### Data collection

**Table:**

Bruker SMART 1000 CCD diffractometer	3354 independent reflections
Radiation source: fine-focus sealed tube	2555 reflections with *I* > 2σ(*I*)
graphite	*R*_int_ = 0.023
ω scans	θ_max_ = 27.0°, θ_min_ = 1.6°
Absorption correction: multi-scan (*SADABS*; Sheldrick, 2004)	*h* = −16→19
*T*_min_ = 0.904, *T*_max_ = 0.944	*k* = −7→7
7882 measured reflections	*l* = −21→15

### Refinement

**Table:**

Refinement on *F*^2^	Primary atom site location: structure-invariant direct methods
Least-squares matrix: full	Secondary atom site location: difference Fourier map
*R*[*F*^2^ > 2σ(*F*^2^)] = 0.038	Hydrogen site location: inferred from neighbouring sites
*wR*(*F*^2^) = 0.109	H-atom parameters constrained
*S* = 1.08	*w* = 1/[σ^2^(*F*_o_^2^) + (0.0601*P*)^2^ + 0.2665*P*] where *P* = (*F*_o_^2^ + 2*F*_c_^2^)/3
3354 reflections	(Δ/σ)_max_ = 0.003
194 parameters	Δρ_max_ = 0.33 e Å^−^^3^
0 restraints	Δρ_min_ = −0.34 e Å^−^^3^

### Special details

**Table:**

Geometry. All e.s.d.'s (except the e.s.d. in the dihedral angle between two l.s. planes) are estimated using the full covariance matrix. The cell e.s.d.'s are taken into account individually in the estimation of e.s.d.'s in distances, angles and torsion angles; correlations between e.s.d.'s in cell parameters are only used when they are defined by crystal symmetry. An approximate (isotropic) treatment of cell e.s.d.'s is used for estimating e.s.d.'s involving l.s. planes.
Refinement. Refinement of *F*^2^ against ALL reflections. The weighted *R*-factor *wR* and goodness of fit *S* are based on *F*^2^, conventional *R*-factors *R* are based on *F*, with *F* set to zero for negative *F*^2^. The threshold expression of *F*^2^ > σ(*F*^2^) is used only for calculating *R*-factors(gt) *etc*. and is not relevant to the choice of reflections for refinement. *R*-factors based on *F*^2^ are statistically about twice as large as those based on *F*, and *R*- factors based on ALL data will be even larger.

### Fractional atomic coordinates and isotropic or equivalent isotropic displacement parameters (Å^2^)

**Table:**

	*x*	*y*	*z*	*U*_iso_*/*U*_eq_	
S1	0.90482 (3)	0.20760 (7)	0.10019 (2)	0.02108 (14)	
C1	0.78421 (10)	0.5823 (3)	0.90053 (9)	0.0194 (3)	
C2	0.80908 (11)	0.4025 (3)	0.86141 (10)	0.0221 (4)	
C3	0.74104 (12)	0.2913 (3)	0.80842 (10)	0.0268 (4)	
H3	0.7557	0.1687	0.7802	0.032*	
C4	0.65245 (12)	0.3572 (3)	0.79640 (11)	0.0299 (4)	
H4	0.6068	0.2791	0.7603	0.036*	
C5	0.62989 (12)	0.5364 (3)	0.83675 (11)	0.0275 (4)	
H5	0.5687	0.5799	0.8279	0.033*	
C6	0.69564 (11)	0.6543 (3)	0.89020 (10)	0.0214 (4)	
C7	0.90521 (12)	0.3284 (3)	0.87383 (11)	0.0290 (4)	
H7A	0.9409	0.4405	0.8547	0.044*	
H7B	0.9077	0.1949	0.8433	0.044*	
H7C	0.9297	0.3017	0.9316	0.044*	
C8	0.67261 (11)	0.8507 (3)	0.93471 (11)	0.0267 (4)	
H8A	0.7064	0.9759	0.9204	0.032*	
H8B	0.6937	0.8256	0.9936	0.032*	
C9	0.57363 (13)	0.9096 (4)	0.91767 (15)	0.0467 (6)	
H9A	0.5525	0.9429	0.8600	0.070*	
H9B	0.5653	1.0361	0.9500	0.070*	
H9C	0.5392	0.7876	0.9320	0.070*	
C10	0.88515 (10)	0.1172 (3)	0.19401 (10)	0.0206 (3)	
C11	0.90431 (11)	0.2524 (3)	0.26121 (10)	0.0246 (4)	
H11	0.9258	0.3948	0.2564	0.029*	
C12	0.89200 (12)	0.1787 (3)	0.33539 (11)	0.0281 (4)	
H12	0.9053	0.2716	0.3812	0.034*	
C13	0.86041 (11)	−0.0297 (3)	0.34369 (11)	0.0266 (4)	
C14	0.84062 (12)	−0.1613 (3)	0.27551 (12)	0.0302 (4)	
H14	0.8180	−0.3028	0.2799	0.036*	
C15	0.85323 (12)	−0.0899 (3)	0.20085 (11)	0.0283 (4)	
H15	0.8401	−0.1825	0.1550	0.034*	
C16	0.84628 (14)	−0.1093 (4)	0.42440 (12)	0.0380 (5)	
H16A	0.8966	−0.0624	0.4677	0.057*	
H16B	0.8430	−0.2675	0.4238	0.057*	
H16C	0.7900	−0.0497	0.4340	0.057*	
N1	0.85616 (9)	0.6956 (2)	0.95856 (8)	0.0200 (3)	
H1A	0.8349	0.8242	0.9725	0.030*	
H1B	0.9036	0.7195	0.9352	0.030*	
H1C	0.8743	0.6129	1.0037	0.030*	
O1	0.88551 (8)	0.43864 (19)	0.09615 (7)	0.0257 (3)	
O2	0.84407 (8)	0.0861 (2)	0.03804 (7)	0.0307 (3)	
O3	0.99931 (8)	0.1642 (2)	0.10318 (8)	0.0311 (3)	

### Atomic displacement parameters (Å^2^)

**Table:**

	*U*^11^	*U*^22^	*U*^33^	*U*^12^	*U*^13^	*U*^23^
S1	0.0223 (2)	0.0194 (2)	0.0217 (2)	0.00109 (16)	0.00531 (16)	−0.00141 (17)
C1	0.0208 (8)	0.0203 (8)	0.0165 (8)	−0.0022 (6)	0.0031 (6)	0.0025 (6)
C2	0.0273 (9)	0.0191 (8)	0.0200 (8)	0.0006 (7)	0.0055 (7)	0.0029 (7)
C3	0.0354 (10)	0.0221 (9)	0.0236 (9)	−0.0033 (7)	0.0078 (7)	−0.0031 (7)
C4	0.0294 (10)	0.0329 (10)	0.0253 (9)	−0.0110 (8)	0.0019 (7)	−0.0032 (8)
C5	0.0217 (9)	0.0322 (10)	0.0276 (9)	−0.0028 (7)	0.0037 (7)	0.0022 (8)
C6	0.0226 (8)	0.0227 (9)	0.0191 (8)	−0.0006 (7)	0.0051 (6)	0.0040 (7)
C7	0.0310 (10)	0.0255 (10)	0.0307 (10)	0.0039 (7)	0.0072 (8)	−0.0050 (8)
C8	0.0208 (9)	0.0275 (9)	0.0313 (10)	0.0002 (7)	0.0047 (7)	−0.0035 (8)
C9	0.0288 (11)	0.0464 (13)	0.0616 (15)	0.0094 (10)	0.0029 (10)	−0.0170 (11)
C10	0.0187 (8)	0.0204 (8)	0.0232 (8)	0.0015 (6)	0.0055 (6)	−0.0007 (7)
C11	0.0241 (9)	0.0231 (9)	0.0267 (9)	−0.0039 (7)	0.0062 (7)	−0.0028 (7)
C12	0.0276 (9)	0.0316 (10)	0.0252 (9)	−0.0024 (8)	0.0060 (7)	−0.0048 (8)
C13	0.0203 (9)	0.0315 (10)	0.0294 (9)	0.0036 (7)	0.0086 (7)	0.0040 (8)
C14	0.0331 (10)	0.0208 (9)	0.0390 (11)	−0.0013 (7)	0.0132 (8)	0.0027 (8)
C15	0.0328 (10)	0.0224 (9)	0.0312 (10)	−0.0029 (7)	0.0102 (8)	−0.0051 (8)
C16	0.0379 (11)	0.0462 (12)	0.0331 (11)	0.0019 (9)	0.0148 (9)	0.0071 (9)
N1	0.0186 (7)	0.0212 (7)	0.0197 (7)	0.0017 (6)	0.0031 (5)	−0.0007 (6)
O1	0.0323 (7)	0.0203 (6)	0.0238 (6)	0.0022 (5)	0.0050 (5)	0.0008 (5)
O2	0.0375 (7)	0.0285 (7)	0.0248 (7)	−0.0043 (6)	0.0044 (5)	−0.0072 (5)
O3	0.0250 (7)	0.0335 (8)	0.0374 (7)	0.0061 (5)	0.0125 (5)	0.0071 (6)

### Geometric parameters (Å, °)

**Table:**

S1—O2	1.4482 (12)	C8—H8B	0.9900
S1—O3	1.4557 (13)	C9—H9A	0.9800
S1—O1	1.4584 (12)	C9—H9B	0.9800
S1—C10	1.7709 (17)	C9—H9C	0.9800
C1—C2	1.390 (2)	C10—C15	1.385 (2)
C1—C6	1.396 (2)	C10—C11	1.390 (2)
C1—N1	1.477 (2)	C11—C12	1.387 (2)
C2—C3	1.393 (2)	C11—H11	0.9500
C2—C7	1.505 (2)	C12—C13	1.395 (2)
C3—C4	1.382 (3)	C12—H12	0.9500
C3—H3	0.9500	C13—C14	1.389 (3)
C4—C5	1.386 (3)	C13—C16	1.512 (2)
C4—H4	0.9500	C14—C15	1.392 (3)
C5—C6	1.397 (2)	C14—H14	0.9500
C5—H5	0.9500	C15—H15	0.9500
C6—C8	1.512 (2)	C16—H16A	0.9800
C7—H7A	0.9800	C16—H16B	0.9800
C7—H7B	0.9800	C16—H16C	0.9800
C7—H7C	0.9800	N1—H1A	0.9100
C8—C9	1.517 (2)	N1—H1B	0.9100
C8—H8A	0.9900	N1—H1C	0.9100
			
O2—S1—O3	113.45 (8)	C8—C9—H9A	109.5
O2—S1—O1	112.66 (7)	C8—C9—H9B	109.5
O3—S1—O1	111.75 (7)	H9A—C9—H9B	109.5
O2—S1—C10	106.17 (8)	C8—C9—H9C	109.5
O3—S1—C10	105.92 (7)	H9A—C9—H9C	109.5
O1—S1—C10	106.21 (7)	H9B—C9—H9C	109.5
C2—C1—C6	123.69 (15)	C15—C10—C11	120.17 (16)
C2—C1—N1	117.11 (14)	C15—C10—S1	120.01 (13)
C6—C1—N1	119.15 (14)	C11—C10—S1	119.79 (13)
C1—C2—C3	117.31 (15)	C12—C11—C10	119.79 (16)
C1—C2—C7	122.52 (15)	C12—C11—H11	120.1
C3—C2—C7	120.16 (16)	C10—C11—H11	120.1
C4—C3—C2	120.86 (16)	C11—C12—C13	120.95 (17)
C4—C3—H3	119.6	C11—C12—H12	119.5
C2—C3—H3	119.6	C13—C12—H12	119.5
C3—C4—C5	120.35 (16)	C14—C13—C12	118.31 (16)
C3—C4—H4	119.8	C14—C13—C16	120.74 (17)
C5—C4—H4	119.8	C12—C13—C16	120.94 (17)
C4—C5—C6	121.05 (16)	C13—C14—C15	121.31 (17)
C4—C5—H5	119.5	C13—C14—H14	119.3
C6—C5—H5	119.5	C15—C14—H14	119.3
C1—C6—C5	116.73 (15)	C10—C15—C14	119.45 (17)
C1—C6—C8	121.31 (15)	C10—C15—H15	120.3
C5—C6—C8	121.96 (15)	C14—C15—H15	120.3
C2—C7—H7A	109.5	C13—C16—H16A	109.5
C2—C7—H7B	109.5	C13—C16—H16B	109.5
H7A—C7—H7B	109.5	H16A—C16—H16B	109.5
C2—C7—H7C	109.5	C13—C16—H16C	109.5
H7A—C7—H7C	109.5	H16A—C16—H16C	109.5
H7B—C7—H7C	109.5	H16B—C16—H16C	109.5
C6—C8—C9	115.46 (15)	C1—N1—H1A	109.5
C6—C8—H8A	108.4	C1—N1—H1B	109.5
C9—C8—H8A	108.4	H1A—N1—H1B	109.5
C6—C8—H8B	108.4	C1—N1—H1C	109.5
C9—C8—H8B	108.4	H1A—N1—H1C	109.5
H8A—C8—H8B	107.5	H1B—N1—H1C	109.5
			
C6—C1—C2—C3	0.8 (2)	O2—S1—C10—C15	27.27 (16)
N1—C1—C2—C3	178.16 (14)	O3—S1—C10—C15	−93.63 (15)
C6—C1—C2—C7	−179.91 (16)	O1—S1—C10—C15	147.40 (13)
N1—C1—C2—C7	−2.5 (2)	O2—S1—C10—C11	−154.60 (13)
C1—C2—C3—C4	−0.7 (3)	O3—S1—C10—C11	84.50 (14)
C7—C2—C3—C4	179.95 (16)	O1—S1—C10—C11	−34.46 (15)
C2—C3—C4—C5	0.4 (3)	C15—C10—C11—C12	0.5 (3)
C3—C4—C5—C6	0.0 (3)	S1—C10—C11—C12	−177.65 (13)
C2—C1—C6—C5	−0.5 (2)	C10—C11—C12—C13	−0.1 (3)
N1—C1—C6—C5	−177.76 (14)	C11—C12—C13—C14	−0.7 (3)
C2—C1—C6—C8	179.76 (16)	C11—C12—C13—C16	−179.50 (16)
N1—C1—C6—C8	2.5 (2)	C12—C13—C14—C15	1.1 (3)
C4—C5—C6—C1	0.0 (2)	C16—C13—C14—C15	179.94 (17)
C4—C5—C6—C8	179.83 (17)	C11—C10—C15—C14	−0.1 (3)
C1—C6—C8—C9	−179.87 (17)	S1—C10—C15—C14	178.07 (13)
C5—C6—C8—C9	0.4 (3)	C13—C14—C15—C10	−0.8 (3)

### Hydrogen-bond geometry (Å, °)

**Table:**

*D*—H···*A*	*D*—H	H···*A*	*D*···*A*	*D*—H···*A*
N1—H1C···O1^i^	0.91	1.88	2.7727 (18)	168
N1—H1B···O3^ii^	0.91	1.89	2.7800 (19)	166
N1—H1A···O2^iii^	0.91	1.95	2.7917 (18)	153

Symmetry codes: (i) *x*, *y*, *z*+1; (ii) −*x*+2, −*y*+1, −*z*+1; (iii) *x*, *y*+1, *z*+1.
